# Left renal vein transposition as a treatment of refractory hypertension in nutcracker syndrome

**DOI:** 10.1016/j.jvscit.2025.101880

**Published:** 2025-06-13

**Authors:** Alexandra B. Hofman, Sophie Ooms, Joris I. Rotmans, Joost R. van der Vorst

**Affiliations:** a Department of Surgery, Leiden University Medical Centre, Leiden, the Netherlands; b Department of Internal Medicine, Leiden University Medical Centre, Leiden, the Netherlands

## Abstract

Nutcracker syndrome is a rare condition that describes compression of the left renal vein in between the proximal superior mesenteric artery and the abdominal aorta. Nutcracker syndrome is diagnosed by combining the typical anamnesis with ultrasound findings, demonstrating left renal vein compression. Hypertension, although rare, can be a serious feature of nutcracker syndrome. There has been a long-standing controversy surrounding possible treatment options for nutcracker syndrome. This case report describes a rare case of refractory hypertension that was treated successfully by left renal vein transposition.

Nutcracker syndrome, also known as aortomesenteric left renal vein entrapment syndrome, is a rare condition that describes compression of the left renal vein (LRV) in between the proximal superior mesenteric artery (SMA) and the abdominal aorta.^1^

Nutcracker syndrome is a rare medical condition and is likely often underdiagnosed. Although it can occur at any age, it is thought to peak during the second and third decades of life.^2^^,^^3^ However, its exact prevalence is not well-known. Patients often present with complaints of hematuria and/or left flank pain and possible associated symptoms of pelvic congestion. Other symptoms that may occur are renovascular hypertension, proteinuria, orthostatic hypotension, or renal dysfunction. Sometimes, a (left-sided) varicocele or varices of the vulva or lower extremities may be present.^2^^,^^4^, ^5^, ^6^

Obtaining a diagnosis can be challenging. Nutcracker syndrome is diagnosed by combining the typical anamnesis, ultrasound findings, and cross-sectional imaging demonstrating LRV compression.^7^ Pressure gradients measured by venography; intravascular ultrasound can confirm the diagnosis subsequently.

There has been a long-standing controversy surrounding possible treatment options for nutcracker syndrome. Options include LRV transposition, gonadal vein transposition, conservative management, stent placement in the LRV, transposition of the SMA or autotransplantation of the left kidney and nephrectomy.^8^ This case report describes a rare case of refractory hypertension that was treated successfully by LRV transposition. Patient consent for publication was obtained.

## Case report

Our patient was a 25-year-old man with a known medical history of hypertension, PDD-NOS, and an inguinal hernia. At the age of 6, he underwent a right nephrectomy owing to hydronephrosis after a complicated pyelonephritis. It is not known if there was an anatomical abnormality beforehand that could explain this course of events. The patient first presented in our clinic at the age of 19, after being diagnosed with nutcracker syndrome in a community hospital. He had been treated for flank and back pain combined with hematuria and hypertension, which had existed for years.

**Fig 1:**
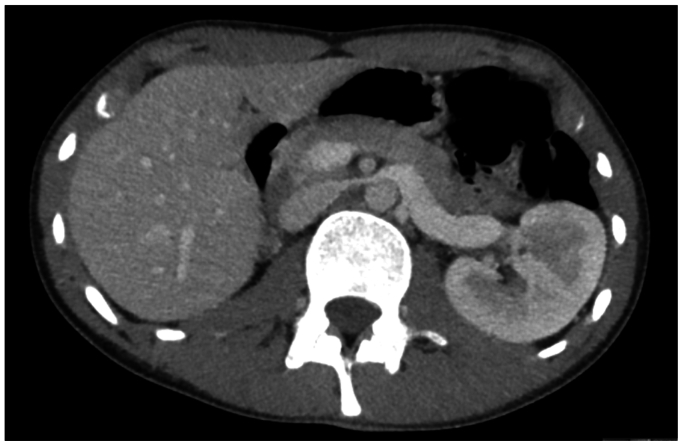
CTA showing compression of the LRV between the superior mesenteric artery (SMA) and the aorta.

On initial presentation to our hospital, our patients' blood pressure was 164/115 mm Hg despite the use of valsartan/hydrochlorothiazide, propranolol, and amlodipine. The more common causes of hypertension were excluded. Potassium, thyroid-stimulating hormone, free thyroxine, cortisol, renin, aldosterone, free normetanephrine, and free 3-methoxythyramin were checked and found to be normal. Genetic testing was performed to check for hypertension- or pseudo-hypoaldosteronism-associated genes. No genetic abnormalities were found. Computed tomographic angiography (CTA) (Fig 1) revealed compression of the LRV between the SMA and the aorta. Venography and duplex ultrasound examination were also obtained. A pressure gradient of 3 mm Hg was recorded, and collateral veins around the renal hilum were seen, further attributing to a nutcracker syndrome diagnosis.

Through a median laparotomy, the LRV was exposed, showing a prestenotic dilatation. The vein was detached from the caval vein and transposed 5 cm caudally. A tension-free end-to-side anastomosis was made. Postoperative recovery was uneventful. Follow-up at the outpatient clinic revealed no abnormalities; the patient remained symptom free and normotensive. Duplex ultrasound examinations were performed at 6 weeks and 1 year after surgery, with both showing no residual compression of the LRV. The peak systolic velocity in the LRV was 0.81 m/s after 1 year. Short-term patency and outcome of the LRV were deemed to be good.

**Fig 2:**
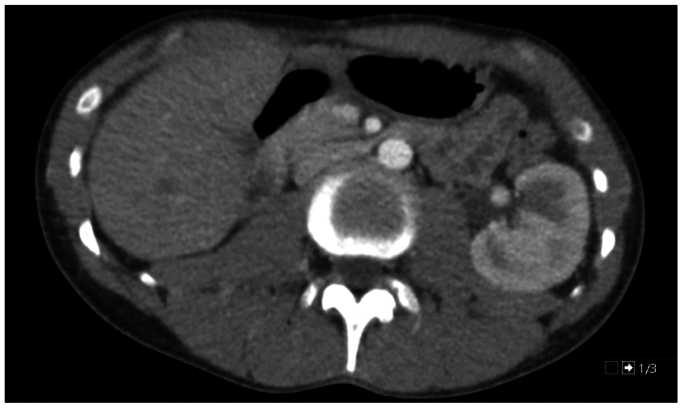
Abdominal CTA revealing tapering of the left renal vein (LRV).

After 2 years, our patient developed microscopic hematuria, intermitting flank pain, and hypertension. In addition, the patient complained of chest pain and an inability to exercise. A CTA of the abdomen showed tapering of the LRV (Fig 2), which was thought to be due to scar tissue or fibrosis. A consecutive venogram was made, showing no pressure gradient. The case was discussed in a multidisciplinary team meeting, ultimately opting for primary conservative treatment.

**Fig 3:**
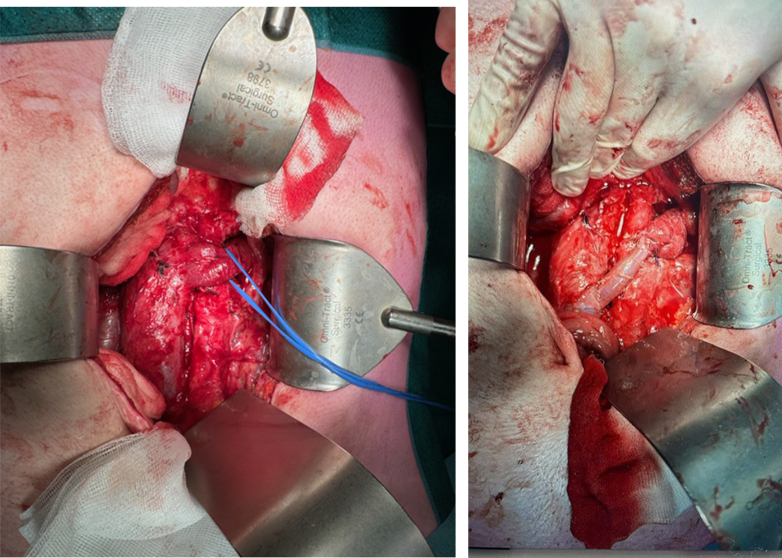
Intraoperative image showing the postoperative result.

The patient complained of exercise intolerance and agitation 1.5 years later. His systolic blood pressures were greater than 200 mm Hg during a 24-hour ambulatory evaluation (Table). This finding was despite four different types of antihypertensive drugs, He displayed persistent microscopic hematuria with a normal creatinine of 88. All other causes of hypertension were excluded. After careful consideration retransposition of the renal vein was performed. Intraoperatively, there some scar tissue was evident, but it was not extensive. It was believed that there might have been a residual compression of the LRV between the SMA and aorta, or a combination of residual compression and scar tissue. The LRV was placed 4.5 cm caudally on the caval vein (Fig 3). A venous interposition was needed to create enough length; this was harvested from the superficial femoral vein to prevent differences in diameter.

**Table:** Twenty-four-hour ambulant blood pressure measurement (conducted on August 15, 2023)

	Total	Day	Night
Systole	219	225	204
Diastole	134	139	122
Mean arterial pressure	173	178	159
Pulse	103	111	80
Pulse pressure	85	86	82

His postoperative course was complicated by hemorrhage and acute kidney injury. A relaparotomy was performed and a hematoma caused by diffuse blood loss was evacuated. The renal vein was patent and had a flow of approximately 200 to 300 mL/min on intraoperative flow measurements. After reintervention, hemoglobin levels stabilized. The acute kidney injury stabilized after 2 days without any intervention other than restricting fluid intake; kidney function recovered completely in the next week. The patient was discharged from the hospital after 9 days.

**Fig 4:**
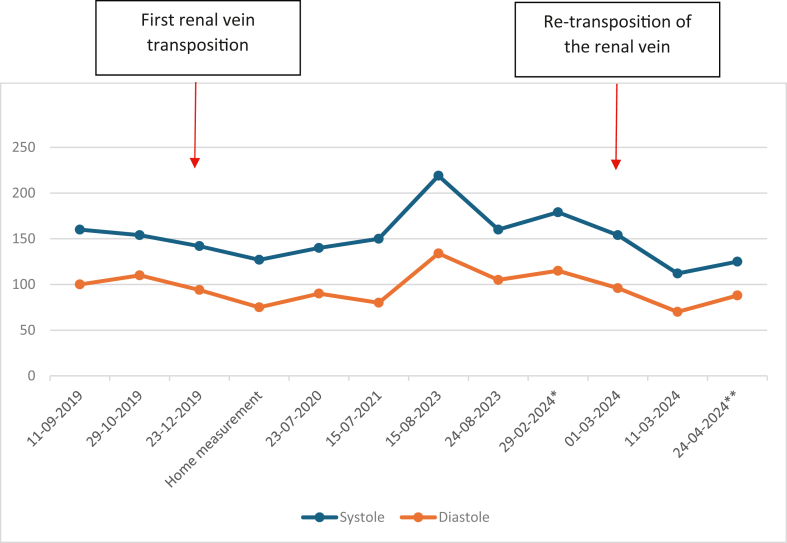
All blood pressure measurements. ∗ Mean value of 50 measurements from December 1, 2023, to February 29, 2024. ∗∗ Mean value of 72 measurements from March 11, 2024, to April 24, 2024.

Remarkably, his blood pressure normalized within days after the surgery. All antihypertensive medications were stopped within weeks. His mean blood pressure was 125/88 mm Hg in a series of 72 consecutive measurements (Fig 4). His creatinine normalized within 3 weeks and has remained stable ever since. Overall, our patient feels fitter, is able to exercise again, and feels less agitated. His complaints of back and flank pain have resolved.

## Discussion

The incidence and prevalence of nutcracker syndrome remain unknown. Compression of the LRV between the SMA and the aorta (the nutcracker phenomenon) is sometimes found on routine CT scans incidentally. However, a large portion of patients with the nutcracker phenomenon do not exhibit a matching symptom pattern. Some patients with this incidental finding are therefore asymptomatic. Hypertension, although rare, can be a serious feature of nutcracker syndrome.

The exact mechanisms by which nutcracker syndrome causes hypertension are unknown, but activation of the sympathetic nervous system and the renin-angiotensin-aldosterone system might play a role.^9^

The diagnostic workup and obtaining a diagnosis can be challenging in nutcracker syndrome. A recently published Delphi consensus document can hopefully aid in the diagnostic process.^7^ In our institution, a duplex ultrasound examination combined with CTA is the first diagnostic step when nutcracker syndrome is suspected. We consider the following duplex criteria presumed in nutcracker syndrome, especially when all are present: an aortomesenteric juxtarenal distance of less than 8 mm, compression of the LRV, high peak systolic velocity within the LRV, and varices.

Hypertension has been described in a handful of case reports,^9^^,^^10^ all with successful remission of hypertension after the invasive treatment of LRV compression. Several therapeutic options are known for the treatment of the LRV compression. Our institution advocates strongly for the renal vein transposition technique, because we believe it offers better long-term durability and carries a lower risk of significant complications compared with stenting, especially in younger patients.

## Conclusions

LRV transposition has proven to be a successful treatment option for therapy-refractory hypertension in nutcracker syndrome. This case report adds to the knowledge and treatment of hypertension as a rare but associated finding in nutcracker syndrome.

## Funding

None.

## Disclosures

None.
